# Positive youth development attributes, mental disorder, and problematic online behaviors in adolescents: a longitudinal study amidst the COVID-19 pandemic

**DOI:** 10.3389/fpubh.2023.1133696

**Published:** 2023-06-02

**Authors:** Xiong Gan, Guo-Xing Xiang, Min Li, Xin Jin, Ke-Nan Qin

**Affiliations:** Department of Psychology, College of Education and Sports Sciences, Yangtze University, Jingzhou, China

**Keywords:** COVID-19, positive youth development, depression, internet gaming disorder, cyberbullying/victimization, Chinese adolescents

## Abstract

**Introduction:**

During the COVID-19 pandemic, adolescents have increasingly suffered from online problem behaviors and mental disorders. But little research has paid attention to the protective factors among adolescents. Therefore, the present study attempted to investigate the role of positive youth development (PYD) attributes in adolescents’ depression, internet gaming disorder (IGD) and cyberbullying/victimization (CBV).

**Methods:**

A total of 995 Chinese adolescents (*M*_age_ = 15.97 years, SD = 0.77, 325 boys) from two public high schools in Hubei province were recruited to participate in the three-wave longitudinal study over the span of 1 year during the pandemic (Time 1: November, 2020; Time 2: May, 2021; Time 3: November, 2021).

**Results:**

T1 PYD attributes negatively predicted T2 depression and T3 online problematic behaviors. T2 depression positively predicted IGD at T3. T3 IGD significantly predicted greater involvement in T3 CBV, and vice versa. Moreover, depression and one online problem behavior mediated the relationships between PYD attributes and the other online problem behavior, separately and sequentially.

**Discussion:**

These findings demonstrated the protective role of PYD attributes in prevention of mental disorders and online problem behaviors among adolescents during the COVID-19 pandemic. Comprehensive measures should be taken to assist young people to develop more PYD attributes to promote healthy growth.

## 1. Introduction

Since it was first reported in Wuhan, Hubei province, at the end of 2019, COVID-19 has dramatically influenced human life in several bursts. During this pandemic, most of the educational, economic, and political activities have to be transferred from offline to online. In the first year of the pandemic, the population of internet users grew by 10.2%, the largest increase in the past decade, and 71% of the worldwide youth (aged between 15 and 24 years) used the internet, which is much higher than other age groups (1). This period of online life is very challenging for individuals, especially for the young generations.

Young people are likely to develop online problem behaviors due to the increased internet availability, such as internet gaming disorder (IGD) and cyberbullying/victimization (CBV) in this specific period. It is well documented that participation in online games and the prevalence of internet gaming disorder have greatly increased among Chinese adolescents (2, 3). Similarly, the prevalence of cyberbullying and victimization has also increased amidst this pandemic (4, 5). These rapid rising trends of online problematic behaviors could be well explained by the perceived COVID-19 impacts such as decreased social connectedness (2, 6). Furthermore, Jessor (7) highlights in the problem behavior theory that problematic behaviors may coexist, and engaging in one problem behavior could promote involvement in another. Adolescents who excessively play online games, especially the violent games, are more likely to bully others or be bullied, and vice versa (8). According to the Neo-ecological Theory, virtual microsystems are critical contexts for youth development in the digital era (9). That is, the online experience is as important as the offline experience and has great effects on individual development. Scholars have revealed that these online problem behaviors will lead to various psychological and physical problems, including damage to brain function (10).

In addition to the online problem behaviors, young people may suffer from more mental disorders during the lockdown, such as depression (2, 11). As one of the most common mental health problems in young generations, depression enjoys a high global prevalence (25.2%) during the COVID-19 outbreak, which is double of the pre-pandemic prevalence (12). Empirical studies indicated that perceived stress of the pandemic and exposure to COVID-19 were positively correlated with depression symptoms (13, 14). Individuals with depression often fail to satisfy their needs in the real world and tend to convert to a virtual world, making them more likely to use the internet, and in turn develop online problematic behaviors. First, the uses and gratifications theory suggests that it is physical and psychological needs that arouse adolescents’ motivations for engagement in online games, and adolescents will obtain a sense of satisfaction in the process of playing online games (15). A meta-analysis revealed that it was more possible for adolescents who suffered from depression and depressive symptoms to develop IGD (16). Second, the psychological decompensation model demonstrates that participation in online activities could compensate individuals for offline psychological developmental difficulties (17). That is, children and adolescents with depression will experience offline psychological problems, which make them develop greater involvement in CBV to seek for pathological compensation. A longitudinal study reported that depression could predict a higher risk of cyberbullying engagement, more difficulty getting out of cyberbullying, and, more seriously, facilitate the transformation of uninvolved adolescents into victims (18). Overall, compared to the healthy counterparts, adolescents who suffer from depression tend to develop greater engagement in IGD and CBV (2, 19).

Given the prevalence, consequences, and development trends of these online problematic behaviors and a mental disorder, it is necessary to obtain a deep and thorough understanding of their antecedents and potential mechanisms prior to formal interventions, where scholars have made many achievements in previous studies. Personality traits and other adverse behavioral factors could explain the reasons for depression (20). And earlier studies have found that cognitive behavioral therapy and interpersonal therapy are effective psychotherapies for depression among adolescents (21). As for the online problematic behaviors, empirical studies have revealed that personal and situational factors will lead to involvement in cyberbullying (22). Various anti-cyberbullying interventions, such as cognitive behavioral programs, have been developed based on risk factors (23). Similarly, internet gaming disorder could also be explained by various family and personal factors, and different interventions, including manualized cognitive behavioral therapy, were applied to the clinical programs (24). However, it is worthwhile to note that there are several research gaps to be addressed. First, few studies have investigated the associations between various online problematic behaviors, which, as mentioned before, could coexist and affect each other. Examining the comprehensive relations could contribute to developing joint intervention programs to prevent or reduce the prevalence of online problematic behaviors. Second, compared with studies on older youth, few studies have been conducted among early and middle adolescents due to the COVID-19 lockdown (11). But as reviewed above, the prevalence of their online problematic behaviors and mental disorder increased dramatically during the pandemic, which implies the urgency and necessity of related studies. Third, most of the previous studies have paid more attention to the risk factors for those mental disorder and online problematic behaviors and neglected to investigate the potential protective factors, especially those personal positive attributes. In fact, adolescents indeed have the strength to promote positive development and prevent negative outcomes (25).

When it comes to adolescents’ strength, positive youth development (PYD) theory provides a widely adopted framework, which summarizes most of the positive attributes that young people have to facilitate thrive (25). Based on this framework, more and more PYD models are gradually developed, including the 5Cs, 6Cs, and 7Cs models, developmental assets model, and the German youth resources model (26, 27). These PYD models are universally used in empirical studies among worldwide youth, including Chinese adolescents (11, 27, 28). One common feature of most models is that they divide the positive factors in adolescent development into two groups: personal resources and environmental resources. According to the PYD framework, there are two basic perceptions: one is the effect assumption, which demonstrates that PYD attributes can effectively foster youth thriving and hinder developmental problems; the other is the cumulative effect notion, which highlights that the more types and higher quality of the positive factors adolescents experience, the more effective the above effects will be (25). Comply with these assumptions, extending research have claimed that PYD attributes are significantly conducive to well-being and thriving, and detrimental to mental disorder and behavioral problems (27, 29). Obviously, most of these findings are reported in the studies conducted during normal periods. Few scholars have evaluated the effect of PYD attributes in specific periods, such as the COVID-19. When a public emergency occurs, it is too difficult for individuals to take objective measures to avoid risks, but positive factors, especially personal attributes, can function to protect individuals. For instance, Shek et al.’s longitudinal study (11) confirmed that PYD attributes could not only negatively predict PTSD symptoms but also buffer the adverse effect of the perceived threat of COVID-19 on PTSD symptoms. Similarly, Xiang and his colleagues (30) verified the protective effects of both personal and social resources in a longitudinal study during the pandemic. Taken together, it is encouraged by theoretical and practical needs to investigate the protective effects of PYD attributes on adolescent mental disorder and online problematic behaviors.

Although there is a lack of literature on the effect of PYD attributes on mental disorder and online problem behaviors during the pandemic, several prior studies have provided indirect evidence of the aforesaid associations in normal periods. As for mental disorder, the latest study has indicated that PYD attributes were negatively related to PTSD symptoms (11). Both cross-sectional and longitudinal evidence suggest that PYD attributes could contribute to the prevention of depression for adolescents (29, 31). Moreover, previous studies have suggested that PYD attributes are beneficial to mental health (27, 32). In terms of problem behaviors, Sun and Shek (33) have confirmed the protective role of PYD attributes in the prevention of problem behaviors, including substance abuse and delinquency. Moreover, abundant studies have revealed that adolescents who have more PYD attributes are less likely to suffer from internet addiction, social networking addiction, internet gaming disorder, and cyberbullying (34–37). In addition, the traditional ecological systems theory claims that individual growth is substantially shaped by the various subsystems (38). The PYD attributes are also from various subsystems such as family, school, community, and individual (25). According to the mindsponge theory, the mind is an information collection-*cum*-processor that can update conditional on environmental conditions and psychological states (39). Adolescents with more PYD attributes from various subsystems will develop high processing power minds, which help them deal with the psychological and behavioral developmental problems and adapt to the changing environment during the COVID-19 pandemic. As a result, it is reasonable to believe that PYD attributes will have protective effects on mental disorder and online problem behaviors during the current specific period.

**Figure 1:**
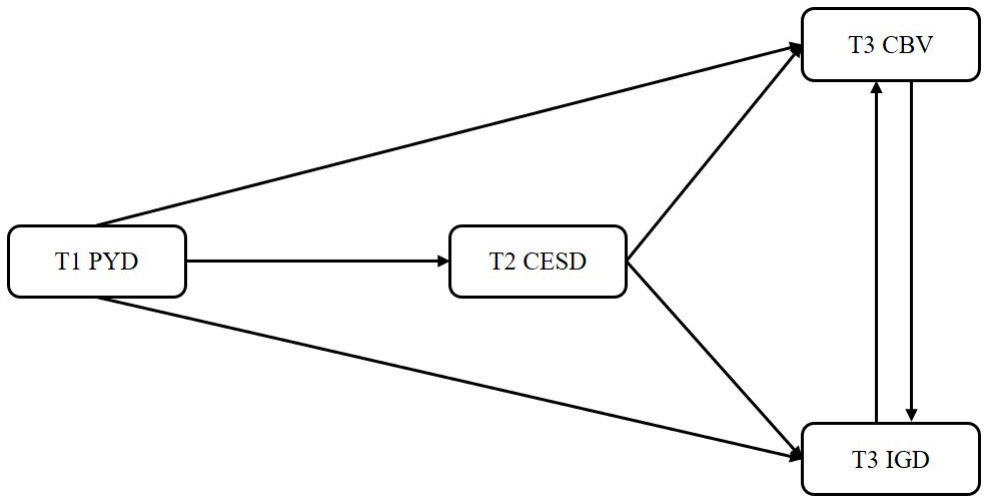
The conceptual longitudinal model. PYD = Positive youth development attributes; CESD = Depression; CBV = Cyberbullying/victimization; IGD = Internet gaming disorder; T1 = Time 1, T2 = Time 2, T3 = Time 3.

Considering the above literature review about the possible relations between PYD attributes, mental disorder and online problem behaviors, the present study aimed to address several research gaps by further exploring the aforesaid associations during the COVID-19 pandemic. Specifically, employing a longitudinal design, we attempted to investigate the potential protective role of PYD attributes in mental disorder and online behavioral problems among Chinese adolescents, which may contribute to the related prevention and intervention. Accordingly, we proposed several hypotheses as follows (Figure 1):

*Hypothesis 1*. Based on the positive youth development theory (25), the traditional ecological systems theory (38), and the mindsponge theory (39), we hypothesized that PYD attributes would inversely predict depression (H1a), CBV (H1b), and IGD (H1c).

*Hypothesis 2*. Considering the uses and gratifications theory (15) and the psychological decompensation model (17), we hypothesized that depression would be positively associated with CBV (H2a) and IGD (H2b) and mediate the relationships between PYD attributes, CBV (H2c), and IGD (H2d).

*Hypothesis 3*. According to the problem behavior theory (7), we hypothesized that CBV would be positively related to IGD (H3a); CBV would mediate the relationship between PYD attributes and IGD (H3b); and IGD would mediate the relationship between PYD attributes and CBV (H3c).

*Hypothesis 4*. We further hypothesized that depression and CBV will sequentially mediate the association between PYD attributes and IGD (H4a); and depression and IGD will sequentially mediate the association between PYD attributes and CBV (H4b).

## 2. Method

### 2.1. Participants and procedure

Adopting a convenience sampling method, adolescents were recruited from two public high schools in Jingzhou, Hubei province, Central China. Students were tenth and eleventh graders. Totally, 995 students participated in the first data collection, and 276 dropped out of the follow-up surveys due to various reasons, such as absence. The final sample consisted of 719 adolescents (M_age_ = 15.97 years, SD = 0.77, 325 boys, 453 in Grade 11). Most of the adolescents (76.21%) were from families at the average economic level or above. 14.47% of them were the only children in their families. Moreover, Chi-square test suggested insignificant differences in gender (*χ*^2^_(1)_ = 1.02, *p* = 0.31), age (*χ*^2^_(4)_ = 5.38, *p* = 0.25), family economic level (*χ*^2^_(2)_ = 1.95, *p* = 0.38) and one-child family (*χ*^2^_(1)_ = 0.45, *p* = 0.50) between the remaining sample and the drop-out sample, indicating that the results in this study would not be biased due to attrition.

Data was collected by pencil-and-paper questionnaires in November, 2020 (T1), May, 2021 (T2) and November, 2021 (T3), when the whole society was affected by the COVID-19 pandemic. Prior to the first formal survey, written consents were obtained from the school leaders, parents, and adolescents through explanation of detailed information about the study. Well-trained employees with negative test results of COVID-19 conducted the survey within 50 min during school hours. Adolescents were encouraged to respond honestly by informing the anonymity of the survey. Participants did not receive any gifts for their participation. The procedures of three times surveys were absolutely the same. Ethical approval for the present study was provided by the Research Ethics Committee of the College of Education and Sports Sciences at first author’s university.

### 2.2. Measures

#### 2.2.1. Positive youth development attributes

The Chinese Positive Youth Development Scale (CPYDS) was adopted to assess adolescents’ PYD attributes (11). The CPYDS measures 15 attributes using 90 items, such as social competence, behavioral competence, and spirituality. One example item is “I can manage my emotions when I have conflicts with others.” Response options range from “1 = not at all or rarely” to “6 = extremely or almost always” on a 6-point Likert scale. Higher total scores indicate more types and higher levels of PYD attributes. This scale suggested good reliability and validity in Chinese adolescents in empirical studies (11). In this study, the Cronbach’s Alpha of this scale at T1 was 0.974.

#### 2.2.2. Mental disorder: depression

The Chinese version of the Center for Epidemiological Studies Depression Scale (CESD) was used to measure adolescents’ depressive symptoms (40). It consists of 20 items rating on a 4-point Likert scale (range from “1 = rarely or none of the time” to “4 = almost or all of the time”). One sample item is “I have trouble concentrating on things.” A higher total score of CESD suggests a higher level of depressive symptoms. This scale had high internal consistency in adolescent samples (40). In this study, its Cronbach’s Alpha was 0.806 at T2.

#### 2.2.3. Online problem behavior

*Cyberbullying/victimization.* The Chinese version of the E-Bullying and E-Victimization Scale (E-BVS) was adopted to evaluate adolescents’ online bullying and victimization in the last year (41). This scale composes 12 items, which rates on a 7-point Likert scale (range from 0 = never to 6 = 6 times or more). One example is “How many times did you say mean things about someone using emails, texting, short messages, on a website such as Renren.” Higher total scores indicate the higher levels of cyberbullying/victimization. This measure showed good reliability and validity among Chinese adolescents (41). In this study, the Cronbach’s Alpha was 0.943 at T3.

*Internet gaming disorder.* The Chinese version of the Internet Gaming Disorder Questionnaire was employed to measure adolescents’ IGD (42). This scale has 11 items with response options on a 3-point Likert scale (“1 = never” to “3 = often”). One example is “Would you skip homework to have more time to play online games.” A higher total score reflects a higher level of IGD. This scale demonstrated good reliability and validity in Chinese adolescent sample (42). In this study, its Cronbach’s Alpha was 0.877 at T3.

#### 2.2.4. Demographic covariates

Several demographic factors were assessed at T1 in this study, including adolescent age, gender (1 = boys, 2 = girls), grade, family economic status (1 = under the average level, 2 = equal to the average level, 3 = above the average level) and family type (1 = family with only one child, 2 = family with two or more children).

### 2.3. Analytic plan

SPSS 26.0 and Mplus 8.0 were used to analyze the data. First, descriptive analysis and Pearson correlational analysis were conducted to descript the basic associations of variables. Second, Structural Equation Modeling (SEM) was adopted to construct two observable variable models in Mplus 8.0 to examine the relationship between PYD attributes, mental disorder and online problem behaviors. Several indices including Comparative Fit Index (CFI), Tucker-Lewis Index (TLI), Root Mean Square Error of Approximation (RMSEA), and Standardized Root Mean Residual (SRMR), were employed to evaluate the model fit. Bootstrapped bias-corrected (BC) 95% confidence intervals (CIs) were also calculated using 5,000 re-samplings to test the multiple mediating effects among key variables.

## 3. Results

### 3.1. Primary analyses

Table 1 displays the descriptive results and the Pearson correlations among the main variables. PYD attributes at T1 were negatively associated with depression at T2 and two problematic behaviors at T3 in small effect sizes. Adolescents who obtained less PYD attributes reported greater engagement in mental disorder and problematic behaviors. Depression at T2 was only positively related to IGD at T3. Adolescents who experienced depression engaged in greater IGD 6 months later. The two problematic behaviors were positively associated with each other in small effect sizes. Adolescents who experienced IGD also showed greater engagement in CBV. Moreover, adolescents gender and age at T1 were correlated with PYD attributes at T1 and problematic behaviors at T3 in small but significant effect sizes, so they were statistically controlled in the following analyses.

**Table 1:** The means, standard deviations, range and correlations among the variables.

	Mean	SD	Range	1	2	3	4	5
*Covariates*								
1. Gender			1 ~ 2					
2. T1 Age	15.970	0.765	14 ~ 18	−0.052				
*Key variables*								
3. T1 PYD	4.796	0.652	3 ~ 6	−0.028	0.097^*^			
4. T2 CESD	1.842	0.390	1 ~ 4	0.051	0.019	−0.193^**^		
5. T3 CBV	2.276	6.621	0 ~ 6	−0.113^**^	−0.034	−0.137^**^	0.066	
6. T3 IGD	1.492	1.712	0 ~ 10	−0.290^**^	0.005	−0.298^**^	0.200^**^	0.218^**^

Gender: 1 = girl, 2 = boy; PYD = Positive youth development attributes; CESD = Depression; CBV = Cyberbullying/victimization; IGD = Internet gaming disorder; T1 = Time 1, T2 = Time 2, T3 = Time 3; *p < 0.05, **p < 0.01.

### 3.2. Main analyses

**Figure 2:**
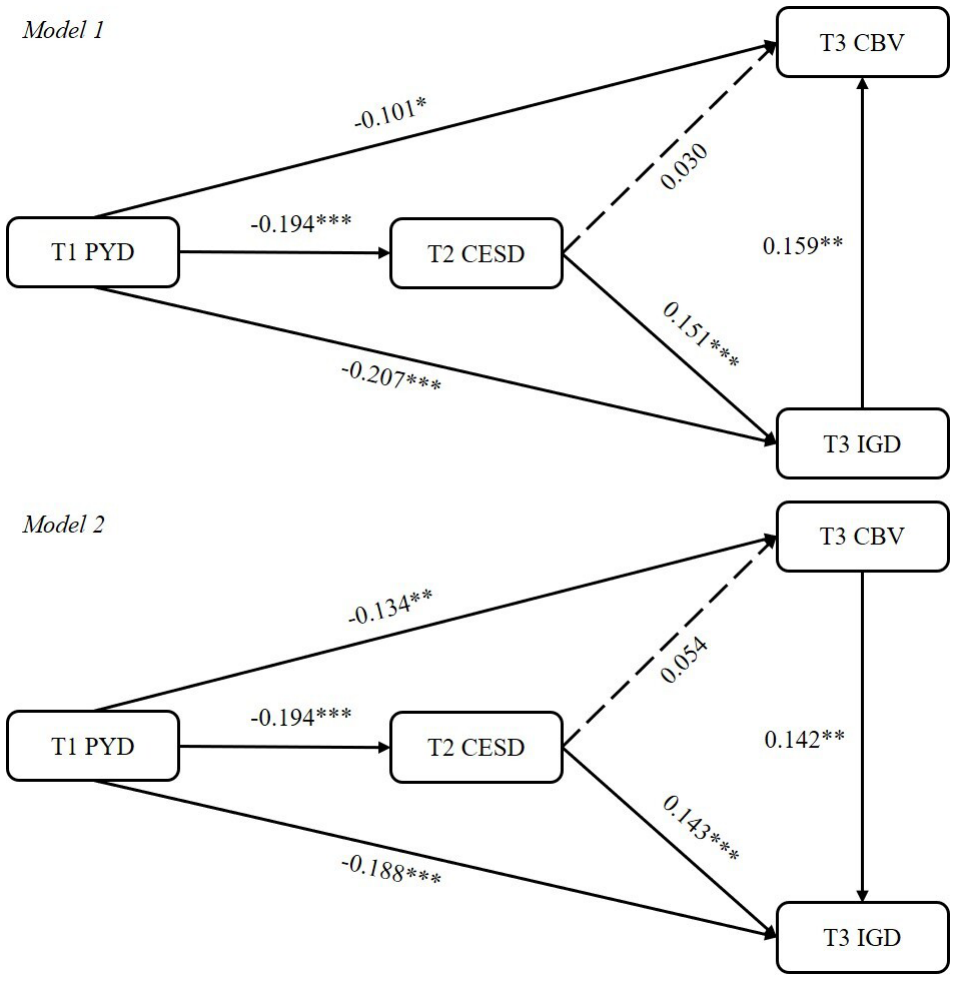
Test results of conceptual longitudinal model. PYD = Positive youth development attributes; CESD = Depression; CBV = Cyberbullying/victimization; IGD = Internet gaming disorder; T1 = Time 1, T2 = Time 2, T3 = Time 3. Adolescent gender and age are covariates; Standardized coefficients are reported; Dashed line indicates a non-significant coefficient. *p < 0.05, **p < 0.01, ***p < 0.001.

Two observable variable models were constructed to examine the predictive effects and mediating effects among the main variables (Figure 2). Both of them were saturation models (*χ*^2^_(0)_ = 0, RMSEA = 0.00, SRMR = 0.00, CFI = 1.00, TLI = 1.00). Table 2 shows the results of regression analysis among the main variables. As expected, PYD attributes at T1 could negatively predict depression at T2 (*β* < 0, *p* < 0.001), T3 CBV (*β* < 0, *p* < 0.05), and T3 IGD (*β* < 0, *p* < 0.001). Adolescents who obtained more PYD attributes would less likely to develop these mental disorder and problematic behaviors. Depression at T2 could positively predict IGD at T3 in a small but significant effect size (*β >* 0, *p <* 0.001). Adolescents experienced depression might suffer from IGD half a year later. IGD at T3 could significantly predict greater involvement in cyberbullying/victimization at the same time point (*β >* 0, *p <* 0.01), and vice versa (*β >* 0, *p <* 0.01). Adolescents would suffer from both two problematic behaviors at the same time or sequentially.

**Table 2:** Test results of the regression analyses.

	T2 CESD			T3 CBV			T3 IGD		
	Estimate	SE	p	Estimate	SE	p	Estimate	SE	p
*Model 1*									
Gender	0.045	0.039	0.245	−0.079	0.037	0.032	−0.271	0.033	0.000
T1 Age	0.149	0.033	0.000	0.032	0.024	0.185	−0.018	0.079	0.822
T1 PYD	−0.194	0.039	0.000	−0.101	0.044	0.021	−0.207	0.046	0.000
T2 CESD				0.030	0.048	0.535	0.151	0.040	0.000
T3 IGD				0.159	0.054	0.003			
*Model 2*									
Gender	0.045	0.039	0.245	−0.122	0.033	0.000	−0.253	0.033	0.000
T1 Age	0.149	0.033	0.000	0.029	0.024	0.232	−0.022	0.077	0.778
T1 PYD	−0.194	0.039	0.000	−0.134	0.040	0.001	−0.188	0.044	0.000
T2 CESD				0.054	0.053	0.307	0.143	0.038	0.000
T3 CBV							0.142	0.046	0.002

PYD = Positive youth development attributes; CESD = Depression; CBV = Cyberbullying/victimization; IGD = Internet gaming disorder; T1 = Time 1, T2 = Time 2, T3 = Time 3. Standardized coefficients are reported.

Table 3 suggests the test results of mediating effects in the two models. Depression at T2 only mediated the relationship between PYD attributes at T1 and IGD at T3 (*β_1_ = −*0.029, *SE_1_ =* 0.010, *95% CI:* [−0.054, −0.014]; *β_2_ = −*0.028*, SE_2_ =* 0.010, *95% CI:* [−0.052, −0.013]). Adolescents who experienced more PYD attributes would less likely to suffer from depression, and in turn they would not develop IGD. T3 IGD could significantly mediate the association between T1 PYD attributes and T3 CBV (*β = −*0.033, *SE =* 0.016*, 95% CI:* [−0.072, −0.012]). Similarly, T3 CBV significantly mediate the pathway from T1 PYD attributes to T3 IGD (*β = −*0.019, *SE =* 0.008*, 95% CI:* [−0.039, −0.007]). Adolescents with more PYD attributes would less likely to develop comorbid problem behaviors. Moreover, depression at T2 and IGD at T3 sequentially mediated the relationship between PYD attributes at T1 and CBV at T3 (*β = −*0.005*, SE =* 0.003*, 95% CI:* [−0.012, −0.001]). Adolescents who develop more PYD attributes early would face less risks of depression later, and in turn develop less involvement in IGD, which then would prevent or reduce engagement in CBV.

**Table 3:** Summary of the indirect effects.

	Estimate	SE	95% CI
*Model 1*			
T1 PYD → T2 CESD → T3 IGD	−0.029	0.010	[−0.054, −0.014]
T1 PYD → T2 CESD → T3 CBV	−0.006	0.010	[−0.026, 0.012]
T1 PYD → T3 IGD → T3 CBV	−0.033	0.016	[−0.072, −0.012]
T1 PYD → T2 CESD → T3 IGD → T3 CBV	−0.005	0.003	[−0.012, −0.001]
*Model 2*			
T1 PYD → T2 CESD → T3 CBV	−0.010	0.011	[−0.033, 0.009]
T1 PYD → T2 CESD → T3 IGD	−0.028	0.010	[−0.052, −0.013]
T1 PYD → T3 CBV → T3 IGD	−0.019	0.008	[−0.039, −0.007]
T1 PYD → T2 CESD → T3 CBV → T3 IGD	−0.001	0.002	[−0.007, 0.001]

PYD = Positive youth development attributes; CESD = Depression; CBV = Cyberbullying/victimization; IGD = Internet gaming disorder; T1 = Time 1, T2 = Time 2, T3 = Time 3. Standardized coefficients are reported.

## 4. Discussion

During the COVID-19 pandemic, adolescents have increasingly suffered from online problem behaviors and mental disorder. However, few scholars have paid attention to the positive factors that could effectively protect adolescents in the specific period. Fortunately, PYD provides a guiding framework to investigate the protective factors. Based on that, the present study adopted a three-wave longitudinal design to evaluate the role of PYD attributes in adolescents’ mental disorder and online problem behaviors amidst the COVID-19 pandemic. Generally, the results verified our hypotheses that PYD attributes were negatively associated with adolescents’ mental disorder and problematic online behaviors during the pandemic. Details of the current findings will be discussed in the following paragraphs.

To begin with, adolescents’ PYD attributes could negatively predict depression, cyberbullying/victimization, and internet gaming disorder. That is, adolescents who developed more positive attributes early would be less likely to suffer from these mental disorder and online problem behaviors. In the long run, scholars have paid little attention to the positive personal factors and have considered adolescents as “problems to be solved” (35). However, this current finding has confirmed that adolescents are “potential strengths to be tapped” and they indeed have positive personal resources that could prevent them from negative outcomes. Theoretically, this finding extends previous studies by directly revealing the important role of PYD attributes on individual development during the specific period (31, 35). This finding provided longitudinal evidence for the PYD theory that the more PYD attributes young people have, the fewer developmental problems they will suffer from (25). Moreover, this finding also supported the mindsponge theory that individuals with more PYD attributes would develop high processing power minds to better cope with the psychological and behavioral developmental problems (39). Overall, PYD attributes could directly provide an umbrella for the young generations when the environmental conditions changed and threatened the individuals’ healthy development.

Besides, PYD attributes could also influence adolescent development indirectly through mental disorder and online problem behaviors, separately and sequentially. First, depression, as reviewed, significantly mediated the relation between PYD attributes and IGD. Adolescents who developed more positive resources would report less involvement in depression, which in turn would prevent them from IGD. This finding further expands the prior studies on the association between depression and IGD (2). Moreover, this finding also empirically supports the uses and gratifications theory and the psychological decompensation model (15, 17). Adolescents who experienced depression or depressive symptoms would have trouble satisfying their physical and psychological needs in offline world. This encouraged them to participate in online activities to compensate and obtain a sense of satisfaction. Second, CBV and IGD could mutually mediate each other’ s association with PYD attributes. Adolescents who perceived more PYD resources would develop less engagement in one problem behavior, which also predicted less involvement in other types of problem behaviors later. This finding supports the problem behavior theory that engaging in one problem behavior could promote involvement in another (7). Furthermore, this result also encourages joint prevention or intervention projects on problem behaviors among young people. Third, depression and IGD played serial mediating roles in the association between PYD resources and CBV, which provides a deeper understanding of the comprehensive relationship between the PYD attributes and these developmental problems. Similarly, this result implies that practitioners might need to consider joint prevention or intervention programs for both mental disorder and behavioral problems in the future.

With regard to implications, the present study may provide several suggestions for prevention and intervention programs. First, mental disorder and online problem behaviors may coexist and influence each other, so it is important for clinical psychologists and other practitioners to detect the occurrence of these comorbidities and focus on the cascading effects in the process of youth development. Second, the PYD framework provides an effective approach by improving positive resources to prevent mental disorder and online problem behaviors in adolescents. For example, Lee et al. (43) developed a PYD game to intervene in adolescent bullying and cyberbullying. Moreover, combined projects, including improving PYD attributes and avoiding risk factors are also encouraged in practice. Although it is not easy to implement, this kind of combined intervention program may obtain more efficient results considering the cumulative effect. Third, under the tense atmosphere of COVID-19, adolescents with more positive attributes could avoid some adverse influence, while those who did not experience enough positive resources were at high-risk. This demonstrates that enhancing PYD attributes should be conducted in the earlier stage of individual development, so that young people could cope with more emergencies, which is also stressed by a previous study (11).

Additionally, the present study has several unique highlights. First, a longitudinal design was employed in the present study to investigate the predictive relationships between variables, while most of the previous related studies adopted a cross-sectional design, limiting the understanding of the relationships. Second, there is a lack of literature on the PYD of youth in Asia, Africa, and Latin America, so the present study recruited a sample of Chinese adolescents to assess the effects of PYD attributes, which enriched the sample diversity in the PYD literature. Third, the present study combined mental disorder and online problematic behaviors to investigate the cascade effects among them, which could contribute to the future work on joint interventions. Fourth, the present study considered the impacts of COVID-19 and confirmed the protective effects of positive factors on individual development during this specific period.

Despite these contributions and unique features, the present study presents several limitations that suggest directions for future research. Initially, the present study used self-reported questionnaires to collect data, which could not avoid inauthentic responses. In the future, scholars may employ more objective approaches to collect data, such as other-reported questionnaires from parents, teachers, or peers. In terms of the sample, we only recruited adolescents from a province in central China. Actually, there are still development differences in various regions in China, so the current sample might not represent adolescents in other regions. Future research is encouraged to include more diverse samples and conduct cross-cultural research to compare the cultural differences. Additionally, the present study only considered one mental disorder and two online problematic behaviors, but there are other developmental problems among young people. Therefore, it is essential for future studies to further explore the effects of positive factors on other developmental outcomes as well as the potential mediating mechanisms and moderators.

## Data availability statement

The raw data supporting the conclusions of this article will be made available by the authors, without undue reservation.

## Ethics statement

The studies involving human participants were reviewed and approved by the Research Ethics Committee of the College of Education and Sports Sciences Yangtze University. Written informed consent to participate in this study was provided by the participants’ legal guardian/next of kin.

## Author contributions

XG designed the study. ML and XJ collected data. K-NQ and G-XX analyzed the data. G-XX drafted and revised the manuscript. All authors contributed to the article and approved the submitted version.

## Funding

This research was supported by Youth project of Science and Technology Research Plan of Department of Education of Hubei Province in 2020 (Q20201306), the Social Science Fund Project of Yangtze University in 2022 (2022csz03), the Faculty Scientific Fund Project of the College of Education and Sports Sciences of Yangtze University in 2022 (2022JTB01), and the key projects of education science plan of Hubei Province in 2022: Study on the influencing factors and intervention mechanism of non-suicidal self-injurious behaviors in adolescents (2022GA030).

## Conflict of interest

The authors declare that the research was conducted in the absence of any commercial or financial relationships that could be construed as a potential conflict of interest.

## Publisher’s note

All claims expressed in this article are solely those of the authors and do not necessarily represent those of their affiliated organizations, or those of the publisher, the editors and the reviewers. Any product that may be evaluated in this article, or claim that may be made by its manufacturer, is not guaranteed or endorsed by the publisher.
